# Large language model-based evaluation of the impact of gender in medical research

**DOI:** 10.1093/jamiaopen/ooag173

**Published:** 2026-09-10

**Authors:** Michael S Yao

**Affiliations:** Perelman School of Medicine, University of Pennsylvania, Philadelphia, PA 19104, United States

**Keywords:** large language models, bibliometrics, medical research, gender disparities, artificial intelligence

## Abstract

**Objective:**

Gender disparities in academic medicine have been previously reported, but prior analyses have relied on either manual labor or fixed databases of name-gender pairs that fail to generalize across different populations and cultures. The objective of this work is to evaluate the utility of large language models (LLMs) as a potential tool to facilitate systematic bibliometric analysis of academic research trends.

**Methods:**

We introduce an LLM-based pipeline that aggregates gender labels from multiple LLM instances to predict the genders of manuscript authors based on their first names.

**Results:**

Our proposed method outperforms alternative algorithms relying on lookup from finite databases of name-gender pairs, while also offering the scalability to tens of millions of authors that is unfeasible with other manual, human-based methods alone.

**Discussion and Conclusion:**

Our results suggest that LLMs can be a powerful tool to scalably track gender-based trends in academic medical research.

## Introduction

Gender disparities in academic medicine have been extensively documented, with evidence of persistent inequities in authorship, leadership, and recognition across multiple clinical specialties.^1–4^ Prior studies have reported that women remain underrepresented in both first and senior authorship roles across disciplines, with implications for academic advancement and visibility, citation patterns, and grant funding.^5–9^ However, these quantitative investigations in medicine have often relied on manual annotation using data from a small set of journals that do not fully capture the breadth of medical literature (Table S1). This limits our ability to detect large-scale and nuanced trends across academic medicine and its subspecialties.

Understanding the scope and evolution of gender disparities in academic research is crucial to promote equity and guide institutional and policy interventions. Prior work has demonstrated that inequities in authorship are associated with differences in citation counts, journal placement, and overall scholarly impact.^1^^,^^10–12^ Furthermore, the dynamics of authorship composition—such as homogeneity of gender within research teams and how gender may correlate with the downstream impact of research—can evolve over time.

Recent work on large language models (LLMs) offers a new opportunity to address these limitations.^13^^,^^14^ By learning from large corpora of natural language, LLMs have demonstrated strong performance in data extraction and classification tasks, including annotation of structured information from unstructured text.^15–17^ Based on these findings, we hypothesize that LLMs may be able to help automate the labor-intensive task of author gender classification in bibliometric analyses, enabling scalable evaluation across large corpora of manuscripts. Such methods can reduce reliance on manual curation, mitigate individual annotator bias, and facilitate frequent monitoring of trends over time. However, the potential of LLMs to systematically examine gender disparities in medical research has not yet been rigorously explored.

In this work, we investigate how LLMs can be used to conduct a large-scale, retrospective bibliometric analysis of academic medical literature. First, we describe an open-sourced computational pipeline to query public databases from the National Institutes of Health (NIH) for clinical and biomedical research articles. Using our curated dataset of the academic literature, we then propose an LLM-based approach to annotate the genders of authors based on their first names. To validate our method, we report on quantitative metrics of authorship trends and research impact stratified by author gender across a decade of publications from 2015 to 2025, and compare our findings with those of prior studies to further characterize the empirical utility of our proposed approach.

## Methods

This retrospective, observational, cross-sectional study was determined to be not human subjects research because it exclusively used publicly available bibliometric data from PubMed, and did not involve human subjects, patient data, or identifiable private information. Therefore, Institutional Review Board approval of this research was not required per the Common Rule (U.S. Department of Health and Human Services, 45 CFR 46).

### Journal and manuscript data curation

We first sought to determine the set of academic journals whose manuscripts would be included in our study. To this end, we used the Entrez programming utilities application programming interface (API) to query the National Library of Medicine (NLM) catalog database for journals that are both (1) published in English and (2) labeled by an NLM-designated broad subject term related to a “medical specialty,” which refers to either general medicine or a medical subspecialty.^18^ See Table S2 for additional details. Journals that stopped publishing manuscripts prior to 2015 were excluded. We note that journals that discontinued publications during the study window were retained, as they contributed citable output within the period of interest. Downstream longitudinal and year-on-year analyses described below should therefore be interpreted with the understanding that not all journals contribute data across every time point, reflecting the natural variation in journal lifespans.

**Figure 1.**
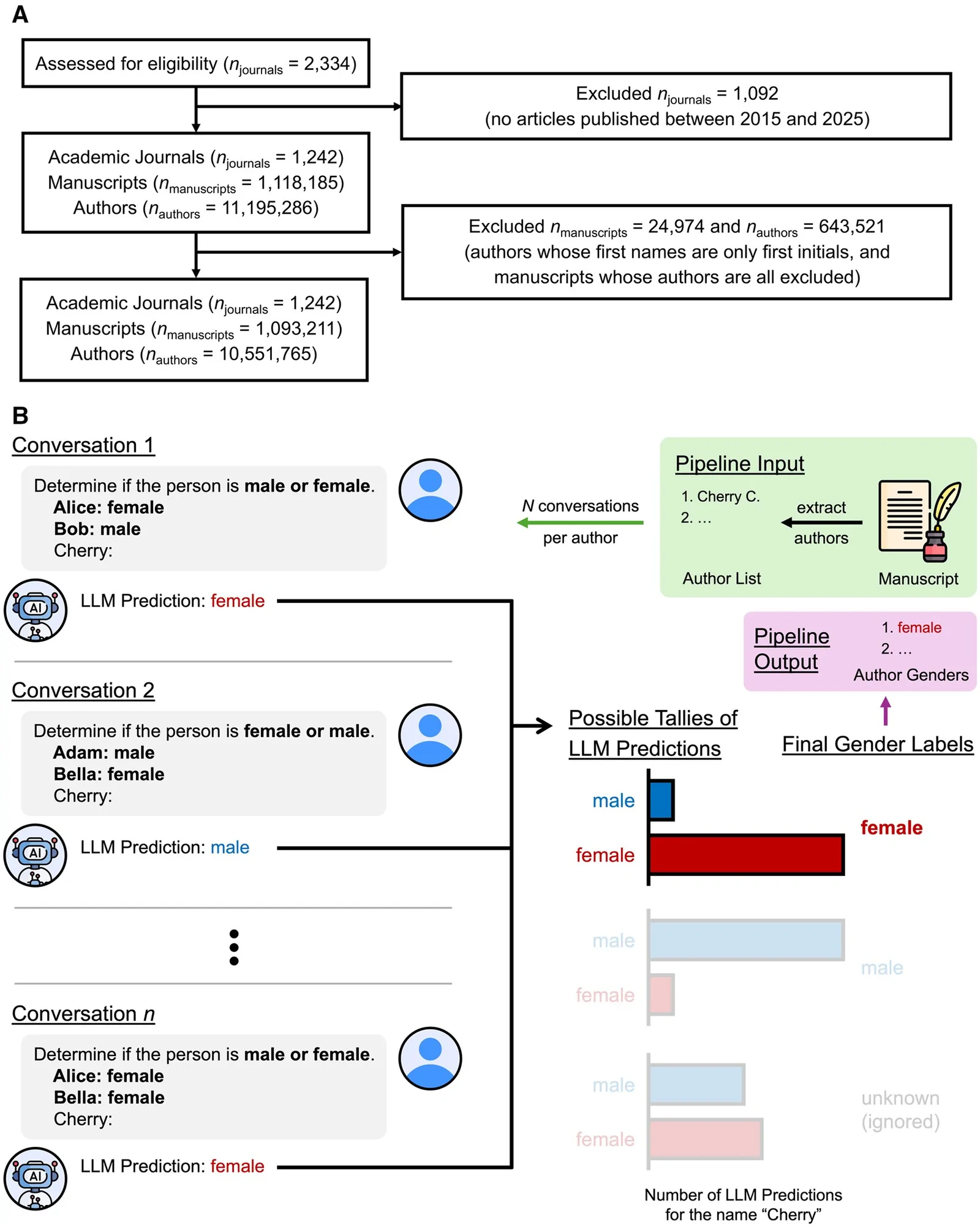
Study flowchart and method overview. (A) Journal, manuscript, and author data were sourced from the National Library of Medicine (NLM) catalog and PubMed Central (PMC); all manuscript data published between January 2015 and September 2025 were included in our analysis. We excluded authors whose first names were only limited to their initials, narrowing the scope of our bibliometric analysis to 1242 unique academic medical journals, 1 093 211 unique manuscripts, and 10 551 765 authors. (B) For each author of a research manuscript, we query a large language model (LLM) to predict the author’s gender from their first name given different semantically equivalent input prompts. Names that were consistently labeled “female” or “male” under different prompt variations were assigned the corresponding gender label.

Using the same Entrez API and a custom Python script, we then queried the PubMed Central (PMC) database for manuscripts published in an NLM-catalogued journal identified above. For each manuscript, we used the API to fetch its author list, year of publication, and digital object identifier (DOI). We restricted our analysis to manuscripts published between January 2015 and September 2025 (Figure 1A). From each author record retrieved from the API, the first name field was extracted directly from the structured API response without any additional string normalization, such that the model’s gender predictions reflected the names as they appear in the published literature.

### Predicting the gender of manuscript authors using a large language model

Prior works analyzing smaller sets of academic manuscripts have almost exclusively used human annotators or fixed-size databases to manually label the gender of manuscript authors.^2^^,^^3^^,^^5–7^^,^^19^ However, such efforts scale poorly and are sensitive to potential internal biases of the annotators.^20^ Furthermore, database-based methods can fail to generalize to previously unseen or rare first names, and may not be able to handle cross-cultural names and account for gender trends that change over time. Recent work has looked to LLMs—which are generative autoregressive models trained on large corpora of text—to effectively automate data annotation pipelines.^21^ Here, we leverage an LLM-based gender annotation approach inspired by contemporary research on leveraging probabilistic sampling and prompt invariance as proxies for the trustworthiness of an LLM’s output.^22–27^ We hypothesize that an accurate LLM gender prediction should be robust to semantically insignificant changes to the input prompt (Figure 1B). Here, we define the integer index s to enumerate the unique author first names in our dataset, and the index i=1, …, n to enumerate the semantically equivalent prompt variants. For a given author’s first name $x_{s}$, we use n=5 different input prompts that phrase the same request using different words to query the Llama-3.1 8B Instruct model from Meta AI (meta-llama/Llama-3.1-8B-Instruct) to predict the gender of the author; see Supplementary Methods for the specific prompts. This process gives us a set of n independently sampled predictions ${\left\{y_{s}^{i}\right\}}_{i=1}^{n}$, where each $y_{s}^{i}\in \{\mathrm{male},\;\mathrm{female}\}$. Using a threshold value of τ=4, we then compute the final label $y_{s}$ according to


ys={male if ∑i=1n1(ysi=male)≥τ female if ∑i=1n1(ysi=female)≥τunknown else 

where 1 is the indicator function that equals 1 if the statement in the function’s argument is true and 0 otherwise. Put simply, the final prediction was “male” or “female” if at least 4 out of the 5 LLM predictions $y_{s}^{i}$ were consistent with one another; otherwise, the final prediction was labeled as “unknown.” We use an LLM temperature parameter of 0.01 for each prediction step; see the Supplementary Methods and Supplementary Results for additional details.

To validate this LLM-based approach, we benchmarked its gender prediction accuracy against baseline methods on 3 publicly available datasets of first names labeled by ground-truth genders: (1) SSA, a dataset of 100 000 unique popular baby names between 1940 and 2024 released by the United States Social Security Administration, (2) Pinyin, a dataset of 96 000 Chinese names from Shi et al.^28^, and (3) Global, a dataset of 4.2 million unique names from Martínez et al.^29^ We compare the performance of our pipeline using Llama-3.1 8B with that of 6 database-based approaches that retrieve gender labels from an exhaustive database.^30–35^ Separately, we also evaluate GPT-OSS 20B (openai/gpt-oss-20b) and 120B (openai/gpt-oss-120b) models using the same LLM-based approach; these are more recently released, state-of-the-art open-source language models compared to Meta Llama-3.1 8B.^36^

### Statistical analysis

To account for multiple comparisons, we used a Bonferroni-corrected significance threshold of *P* < .001 to indicate a statistically significant difference. *P* values less than 2.225 × 10^−308^ (ie, the smallest 64-bit normal float that can be represented in IEEE-754 format) are reported as *P* = .0. Differences between the proportions of male and female authors were quantified using Cohen’s *h* statistic,^37^ where positive (respectively, negative) values denote a greater proportion of male (respectively, female) authors. Differences between the rates of change in authorship by gender over time were quantified using Cohen’s *d*.^38^ For both measures, we follow the convention from prior work of interpreting absolute value thresholds of 0.2, 0.5, and 0.8 as small, medium, and large effect sizes, respectively.^38^^,^^39^ All statistical analyses were performed using Python software (version 3.13.7; Python Software Foundation) and the SciPy scientific computing package (version 1.16.1; NumFOCUS). Data deduplication was performed based on unique identifiers (NLM IDs for journals, PMC IDs for articles, and DOIs for citation analysis) to prevent artificial inflation of sample sizes. All experiments were run on one 96-core Intel Xeon CPU and one NVIDIA RTX A6000 GPU (CUDA version 12.6).

## Results

### Data description

We identified a total of 2334 unique journals in the NLM catalog and filtered them to the subset of 1242 journals that published at least 1 manuscript between January 2015 and September 2025 (Figure 1A). From these journals, approximately 11 million manuscript authors were identified from PMC. We then excluded approximately 650 000 (5.75%) authors because their first names were only initials (making it impossible to reliably determine their gender). Any manuscripts whose authors were all excluded due to the above criteria were also excluded. Our final dataset included 1 093 211 manuscripts and 10 551 765 authors publishing in 1242 unique academic journals. Additional analysis of the distribution of research articles published within each journal is included in Figure S1, and the full dataset of medical research journals included in our study is detailed in Data S2.

### Validation of our LLM gender annotation pipeline

**Figure 2.**
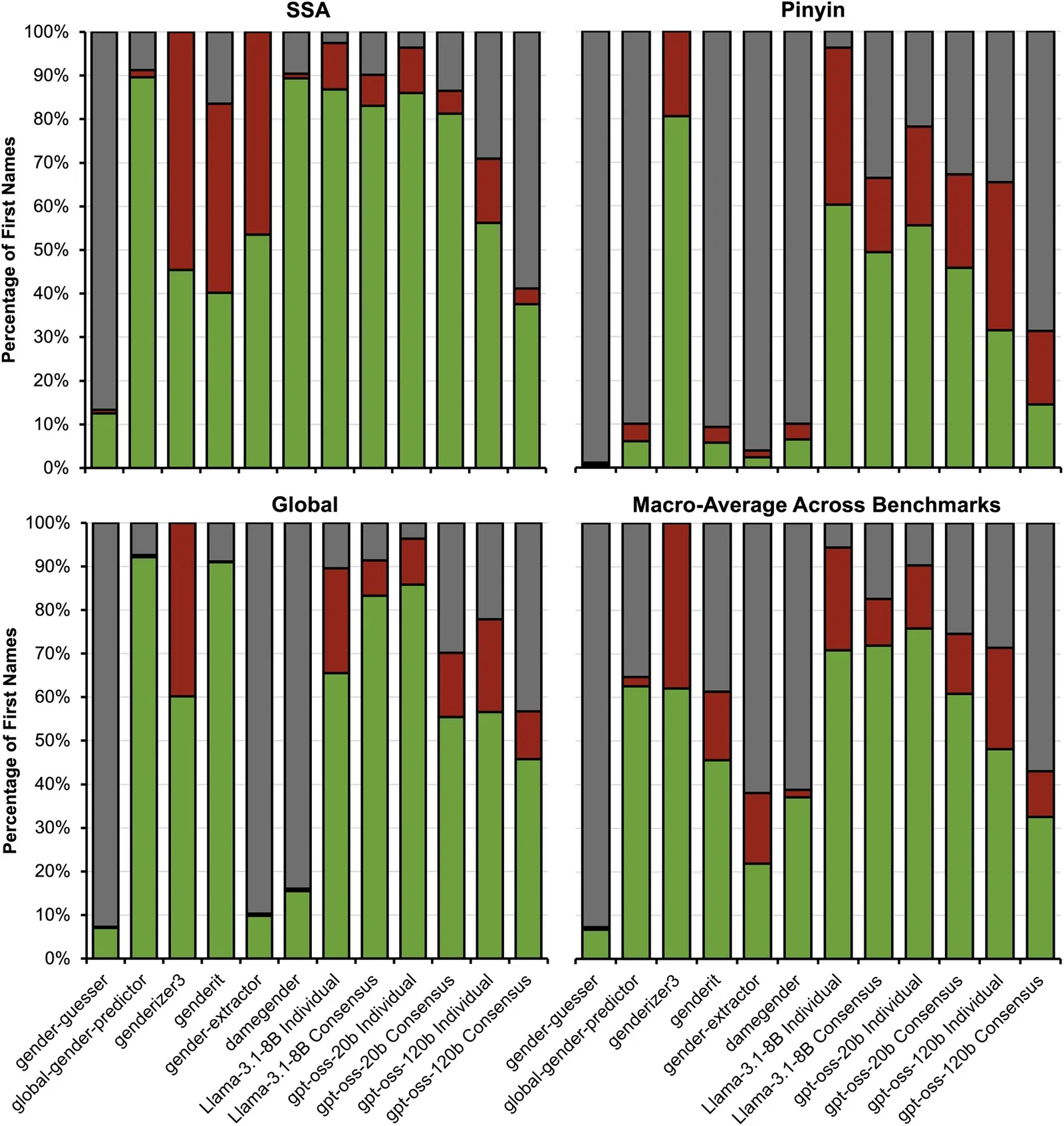
Benchmarking gender annotation methods by first name. We compare the performance of our proposed gender annotation method using Llama-3.1-8B against alternative approaches, including 6 databases that retrieve from a fixed enumeration of name-gender pairs (gender-guesser, global-gender-predictor, genderizer3, genderit, gender-extractor, and damegender), and 2 alternative backbone LLMs to use in our pipeline (gpt-oss-20b and gpt-oss-120b). For the LLM-based methods, “individual” refers to using a single LLM prediction to assign genders, while “consensus” refers to using the aggregate predictions of n=5 independent LLM predictions and a threshold value of τ=4 as described in our Methods section. Green, red, and gray bars refer to correctly labeled, incorrectly labeled, and unknown gender labels, respectively.

We compare our proposed LLM-based gender annotation strategy against alternative approaches in Figure 2. While database-based methods performed reasonably well compared to (and sometimes better than) our proposed LLM-based pipeline on the SSA dataset with ostensibly Western names, we found that they struggled significantly on the Pinyin and Global benchmarks. Macro-averaging across the 3 benchmarks, gpt-oss-20b achieved the greatest percentage of correct gender assignments at 75.8%, followed by Llama-3.1-8B at 70.9%. Across the LLMs evaluated, we found that using our thresholding strategy to aggregate gender predictions across n=5 independent LLM predictions reduced the frequency of incorrect gender predictions while simultaneously increasing the frequency of unknown gender predictions, as expected. This thresholding strategy offered the greatest improvements using Llama-3.1-8B as the backbone LLM, where the macro-averaged percentage of incorrect predictions decreased from 23.6% to 10.8% while the percentage of unknown genders increased from 5.6% to 17.3%. Based on these results, we chose to continue using the Llama-3.1-8B model in downstream experiments. Additional experimental validation of our proposed method is included in Figures S2, S3, and S8 and Tables S3 and S4.

We also compared the gender annotation rate of our LLM-based approach to that of other gender annotation methods used in prior works. We define the gender annotation rate as the proportion of manuscript authors that were able to be assigned a gender of either “male” or “female.” Our method achieved an annotation rate of 85.2% across all specialties, which is comparable to prior work using database-based approaches for gender annotation.^4^^,^^40^^,^^41^ Other works that use manual, human-based annotation methods have achieved higher annotation rates as high as 98.5%;^2^^,^^42^^,^^43^ however, these prior studies also acknowledge that the success of their methods relied on the ability of researchers to search for the gender of the author online based on their public Internet profile. In contrast, web search capabilities were not made available to our LLM-based method.

### Manuscript authorship over time

**Figure 3.**
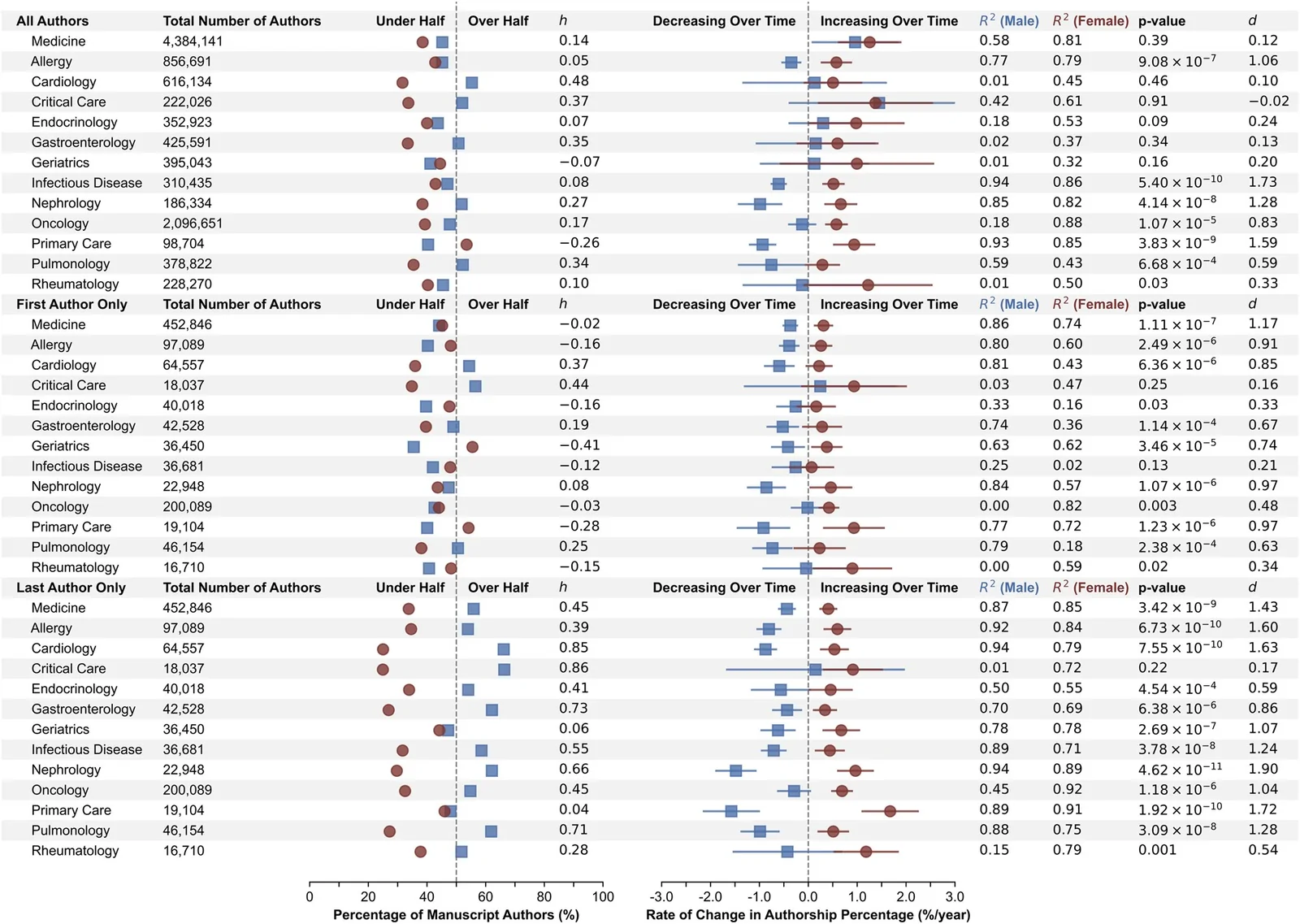
Gender analysis of manuscript authors and authorship trends. (Left) Within each of the 13 medical subjects defined by the National Library of Medicine (NLM), we show a scatter plot of the proportion of (top) all manuscript authors; (middle) first authors; and (bottom) last authors that are predicted to be male (blue squares) or female (red circles) and report the associated Cohen’s *h* effect sizes. (Right) We also fit separate linear regression models to describe how these gender proportions change as a function of publication year. We then plot the estimated rate of change in authorship percentage and report the coefficient of determination (*R^2^*) of each regression model; error bars represent 99.9% confidence intervals of the true parameter values. Finally, we conducted 2-sided, unpaired Wald tests with the Satterthwaite approximation to determine if the rates of change are different between male and female authors within each row; the corresponding *P*-values are listed in the penultimate column and Cohen’s *d* effect sizes are listed in the final column. Reported percentages are with respect to all authors in our dataset (including the authors whose gender could not be determined), meaning that the respective percentages of predicted female and predicted male authors within each row may sum to less than 100%.

To better characterize the genders of manuscript authors using the consensus gender labels predicted by our proposed method, we first computed the fractions of male and female manuscript authors. We found that the proportion of male authors was greater than that of female authors for all evaluated medical specialties except for primary care and geriatrics (Figure 3). However, when focusing on only the first authors of each manuscript, female gender representation was greater in 8 out of the 13 specialties (general medicine, allergy, endocrinology, primary care, infectious disease, geriatrics, oncology, and rheumatology). The last authors of manuscripts were more frequently male than female across all medical subspecialties. Traditionally, the first author is typically the researcher who leads the research work, while the last author is the principal investigator (PI) responsible for the overall integrity of the work.

We then sought to determine if these gender proportions have changed over time. To this end, we estimated separate ordinary least squares regression models to predict the observed proportion of each gender each year within each subspecialty (Figure 3). Broadly, we found that female gender representation was increasing over time with statistical significance in the population of all manuscript authors for 8 out of 13 medical subspecialties; first authors for 6 subspecialties; and last authors for 12 subspecialties. Notably, these results are consistent with prior work describing increasing female representation as PIs of academic research labs.^44^^,^^45^ Additional quantitative results are shown in Figure S4.

## Discussion

Our study contributes to the growing body of literature examining gender disparities within medical research. Previous investigations have largely relied on manual annotation and were constrained by relatively small sample sizes, limiting their ability to capture large-scale trends (Table S1).^5–8^^,^^19^ To this end, our findings demonstrate that LLMs can help scale the systematic evaluation of authorship and citation patterns in academic medicine, enabling broader and more nuanced insights into issues of gender representation in the field.

Using our proposed method, our results in Figure 3 reproduce previously described gender disparities in medical authorship across specialties, with effect sizes that vary meaningfully by authorship position. The differences by gender were consistently largest for last authorship positions, with the largest effects observed in cardiology, critical care, and gastroenterology (Cohen’s *h *≥ 0.73). First authorship disparities were generally more modest (Cohen’s *h *= −0.02 when pooling across all fields of medicine), suggesting that early-career participation by women may be improved relative to leadership roles. However, meaningful progress in female representation in academic research has been made over the past decade, particularly in cardiology, nephrology, and primary care (Cohen’s *d *≥ 1.63). Taken together, these observational findings suggest that the disparity at senior authorship positions, while still large in absolute terms, is narrowing over time.

Our findings highlight both persistent inequities and promising shifts in medical research by gender. Continued structural and cultural interventions are needed to promote equity in authorship, collaboration, and scientific recognition. Importantly, our study also reproduces key findings from prior works analyzing smaller, domain-specific subsets of manuscripts, strengthening the validity of our LLM-based approach for gender-based bibliometric studies.^2^^,^^4^^,^^40^^,^^44^^,^^46^ Our results ultimately demonstrate the utility of LLM-driven analyses for ongoing monitoring of research trends. Future work might examine how to extend our method to evaluate the longitudinal effects of policy initiatives and identify mechanisms that promote equitable participation in medical research.

There are important limitations associated with our work that warrant discussion. Firstly, our findings comprise an observational bibliometric study that is not designed to interrogate potential causal relationships between gender and academic metrics. Our findings only represent observed associations based on our proposed LLM-based gender prediction method. Separately, individual gender identities may not align with LLM-generated predictions; recall that we used the Llama-3.1 8B model from Meta AI (a US-based company) to automate the annotation of gender labels by author first name. We chose to use this particular language model because it is easily accessible on consumer-grade hardware and performed well on the task at hand. However, it may also underperform on authors with first names that are not common in the United States. For example, Chinese first names written in Mandarin often encode gender in the specific Chinese characters used, which is important information that is lost when the name is translated to English. Separately, different subpopulations of authors may have different incidences of unisex names or choose to publish using only first initials. These limitations are exemplified in our results in Figure 2, where almost all gender annotation methods that were evaluated performed worse on the Pinyin and Global datasets compared to the SSA dataset. We further analyze the performance of different methods as a function of geographic region and associated differences in first name frequency in Figure S8. Further discussion of the observed failure modes associated with our LLM-based gender annotation pipeline is included in Table S4. Future work might explore how to use modern multilingual and more performant language models to better close this potential gap,^47^^,^^48^ or leverage more sophisticated LLMs with web search capabilities to leverage additional author metadata, such as full author names, country of affiliation, or institutional metadata, to further improve gender annotation accuracy. Such metadata could also be used to disambiguate individuals who share the same first and last names, which could help power future work on academic collaboration networks, subgroup dynamics, and other bibliometric analyses of interest.

We also note that our current method is not designed to recognize non-binary and gender-diverse individuals who may not identify with the gender most commonly associated with a first name at a population level.^49^ This raises potential ethical and cultural concerns. Firstly, any automated gender annotation method can only impute gender from a statistical association between names and a population-level naming convention. In doing so, it collapses a self-determined, multidimensional identity into a binary gender label. This may inadvertently exclude non-binary, transgender, and intersex individuals who are either misgendered or silently discarded.^50^^,^^51^ To this end, we highlight that our fairness analysis in Figure S8 is only with respect to the binary gender framework assumed in our work. We also found that gender annotation performance was correlated with ethnicity and geography. This implies that the results reported in our work may not be representative of underrepresented minorities in our evaluation dataset.

Furthermore, other variables of interest, such as author nation of origin and institutional affiliation, might be interesting to explore using a similar LLM-based method in follow-up bibliometric analyses. However, a key challenge with this proposed study lies in scalably and accurately determining these variables for large groups of manuscript authors, as prior work has relied on human efforts to laboriously query the Internet for this information on an author-by-author basis. This problem limits our ability to evaluate for potential confounders, such as researcher career stage, institution, and geographic location. However, recent advancements in the web-search and multi-step reasoning capabilities of language models may help address this limitation.^52–54^ One might also use these tools to conduct additional downstream analyses as a function of predicted gender, such as examining how collaboration networks, funding patterns, institutional affiliations, and geographic trends might vary as a function of researcher gender. We leave a rigorous exploration of how to best integrate these technologies into bibliometric analyses as an opportunity for future work.

We also limited our analysis to research available on PubMed, which only contains a subset of the total body of medical research. For example, non-archival materials including conference abstracts, oral presentations, and research posters are typically not deposited in PubMed. Because PubMed is also an archival resource hosted by the United States, our results are inherently Anglocentric and may not accurately represent the bibliometric trends of other areas of the world. Some research teams may also choose not to openly publish their research to protect intellectual property, preserve competitive industry know-how, or a variety of other reasons. Our method is also inherently tied to the accuracy and maintenance of external, third-party resources such as the PMC database. The manuscript coverage of these repositories may be incomplete and vary across both academic fields and time, which may impact the validity of our results. Future work may be warranted to better characterize the real-world significance of this potential limitation.

## Supplementary Material

ooag173_Supplementary_Data

## Data Availability

Raw source data for all figures and all data generated by this study are available at https://doi.org/10.5061/dryad.bvq83bkq5.

## Code availability

We make our custom code implementation publicly available at https://github.com/michael-s-yao/medicine-authorship.
