# Supplementary material for: Large language model-based evaluation of the impact of gender in medical research
Source: JAMIA Open. 2026 Sep 10;9(5):ooag173. doi: 10.1093/jamiaopen/ooag173 (PMC13561525; doi:10.1093/jamiaopen/ooag173)
Supplement: Supplementary file 1 [file ooag173_supplementary_data.docx]

**Supplementary Information**

Large Language Model-Based Evaluation of the Impact

of Gender in Medical Research

Michael S. Yao^1^

**Affiliations**

^1^University of Pennsylvania, Philadelphia, PA, USA 19104

**Corresponding Author:**

Dr. Michael S. Yao

University of Pennsylvania

3330 Walnut St

Philadelphia, PA, USA 19104

Tel: +1(215)898-8560

Email: michael.yao@pennmedicine.upenn.edu

**Table of Contents**

Supplementary Figures 1 – 8 …………………………………………………………………. S1 – S8

Supplementary Tables 1 – 8 …………………………………………………………………. S9 – S16

Supplementary Methods ………………………………………………………………….... S17 – S18

Supplementary Results …………………………………………………………………….. S19 – S24

Supplementary References ………………………………………………………………... S25 – S26

# Supplementary Figures

**Supplementary Figure 1: Distribution of research articles by journal.** For each medical specialty, we plot a Pareto chart of the PubMed Indexed Journals on the *x*-axis sorted by the number of manuscripts published in each journal between January 2015 and September 2025, plotted on the left *y*-axis in solid black. We also plot the cumulative proportion of published manuscripts in each specialty in dotted dark grey on the right *y*-axis. The fraction of journals that contain at least 99% of published manuscripts in the specialty is shown by the dotted-dashed red line. Note that all journals within each specialty have been compressed down to the same *x*-axis “length” – the total absolute count of journals shown varies by specialty.

**Supplementary Figure 2: Gender prediction inaccuracies stratified by true gender.** For each of the gender annotation methods assessed in **Figure 2** in the main text, we report the probability of obtaining a wrong gender label conditioned on the true gender associated with a given first name. We report probabilities as a percentage across the (**left**) SSA (United States Social Security Administration); (**middle**) Pinyin; and (**right**) Global benchmarking datasets. For the LLM-based methods, “Individual” refers to using a single LLM prediction to assign genders, while “Consensus” refers to using the aggregate predictions of $n=5$ independent LLM predictions and a threshold value of $\tau=4$ as described in our **Materials and Methods** in the main text.

**Supplementary Figure 3: Unknown gender predictions stratified by true gender.** For each of the gender annotation methods assessed in **Figure 2** in the main text, we report the probability of assigning a gender label of “unknown” conditioned on the true gender associated with a given first name. We report probabilities as a percentage across the (**left**) SSA (United States Social Security Administration); (**middle**) Pinyin; and (**right**) Global benchmarking datasets. For the LLM-based methods, “Individual” refers to using a single LLM prediction to assign genders, while “Consensus” refers to using the aggregate predictions of $n=5$ independent LLM predictions and a threshold value of $\tau=4$ as described in our **Materials and Methods** in the main text.

**Supplementary Figure 4: Number of authors of medical research papers over time.** The left (resp., right) heatmap shows the number of female (resp., male) authors of research papers in each medical specialty (rows) by year (columns). Note that while our main study considers manuscripts published between January 2015 and September 2025, we do not show the number of authors from the 2025 calendar year, which was incomplete at the time that our study was conducted.

**Supplementary Figure 5: Analysis of gender distributions within manuscript author lists.** For each medical subjects defined by the National Library of Medicine (NLM), we plot the distribution over manuscripts of the percentage of author lists that are predicted to be female (red left side of each violin plot) and male (blue right side of each violin plot). The median of each distribution is indicated by solid black lines, and the first and third quartiles are indicated by dotted black lines. Reported percentages are with respect to all authors of an associated manuscript (including the authors whose gender could not be determined), meaning that the respective percentages of predicted female and predicted male authors for a given manuscript may sum to less than 100%.

**Supplementary Figure 6: Manuscript citation and reference trends as a function of author gender.** We plot the mean and 99.9% confidence intervals of the number of (**left**) total citations; (**middle**) self-citations; and (**right**) total references as a function of the gender of the first and last author. For each medical subspecialty and metric, we also ran a two-sided unpaired *t*-test to determine if the values of each metric are significantly different between first and last authors of different genders within each row; the corresponding *p*-values are listed in the final column.

**Supplementary Figure 7: Gender-based differences in SCImago Journal Rank (SJR) and journal Open Access (OA) status.** (**Left**) For each medical specialty, we first plot the percentage of manuscripts published in OA journals as a function of the gender of the first and last authors. The *p*-values are computed from a two-sample *z*-test to compare the time-averaged marginal effect of gender on the year-over-year proportion of OA manuscripts. (**Middle**) We then fit separate logistic regression models for each specialty and author position to estimate the probability of a manuscript being published in an OA journal as a function of author gender and year, and plot the mean and 99.9% confidence interval of the change in OA log odds per year by gender according to the fitted model. The reported *p*-values are computed from a two-sample, unpaired *t*-test to compare the difference in mean change in OA log odds per year by gender. (**Right**) Finally, we plot the mean and 99.9% confidence interval of the journal SJR as a function of author gender. The reported *p*-values are computed from a two-sample, unpaired *t*-test to compare the difference in mean SJR by gender.

**Supplementary Figure 8: Cross-cultural performance of gender prediction methods.** For each global region and gender prediction method, we report (**A**) the unweighted mean of F1 scores stratified by gender; and (**B**) the within-region difference in mean F1 score by gender, where blue (resp., red) cells correspond to better performance on male (resp., female) names. (**C**) Bar plot comparing the performance of gender prediction methods on Western (Anglosphere, West/North Europe, and South Europe) versus non-Western names.

# Supplementary Tables

| **Publication** | **Number of Authors** | **Number of Manuscripts** | **Number of Journals** | **Gender Annotation Strategy** | **Time Period** | **Annotation Success Rate** |
| --- | --- | --- | --- | --- | --- | --- |
| [Bruns N et al. Crit Care (2025).](https://doi.org/10.1186/s13054-025-05649-4)^1^ | 77,713 | 42,970 | 5 | Proprietary ([NamSor](https://namsor.app/)) | 2005-2024 | 87.3% |
| [Brück O. Nat Commun Med (2023).](https://doi.org/10.1038/s43856-023-00417-3)^2^ | 4,217 | 10,058 | 5 | Database ([GenderizeR](https://github.com/kalimu/genderizeR)) | 2010-2019 | 94.1% |
| [Böhme K et al. Ped Res (2022).](https://doi.org/10.1038/s41390-022-02010-1)^3^ | 363,518 | 156,642 | 400 | Database ([Gendermetrics.Net](https://doi.org/10.1186/s12995-016-0133-6)) | 2008-2018 | 76.0% |
| [Krstacic JE, et al. PLoS One (2022).](https://doi.org/10.1371/journal.pone.0261209)^4^ | 2,900 | 1,080 | 3 | Manual | 2012-2019 | 98.4% |
| [Yao J, et al. J Glob Health Rep (2022).](https://doi.org/10.29392/001c.33616)^5^ | 190,809 | 33,854 | 46 | Database ([Gender-API](https://gender-api.com/)) | 1945-2020 | 74.8% |
| [Chatterjee P, et al. JAMA Netw Open (2021).](https://pubmed.ncbi.nlm.nih.gov/34213560/)^6^ | 10,494 | 5,554 | 5 | Database ([GenderizeR](https://github.com/kalimu/genderizeR)) and Manual | 2015-2018 | 98.1% |
| [Campbell JC, et al. J Am Coll Radiol (2019).](https://pubmed.ncbi.nlm.nih.gov/30722843/)^7^ | 11,657 | 1,934 | 3 | Manual | 2011-2015 | N/A |
| [Jagsi R et al. N Engl J Med (2006).](https://doi.org/10.1056/NEJMsa053910)^8^ | 7,249 | 3,872 | 6 | Manual | 1970, 1980, 1990, 2000, and 2004 | 98.5% |
| **Ours** | 10,551,765 | 1,093,211 | 1,242 | LLM | 2015-2025 | 85.2% |

**Supplementary Table 1: Comparison against prior work.** To motivate our approach in using language models to scale bibliometric analysis, we compare the scale of our analysis against prior work looking at gender disparities in academic medical research. Our approach enables us to analyze an order of magnitude more authors, manuscripts, and journals to gain insights into research trends in academic medicine compared with existing work. Furthermore, our proposed method achieves an annotation success rate that is at least that of other automated methods.

| **Medical Specialty** | **Broad Subject Terms** |
| --- | --- |
| Medicine | [Internal Medicine](http://www.ncbi.nlm.nih.gov/nlmcatalog?term=Internal%20Medicine%5Bst%5D) |
|  | [Medicine](http://www.ncbi.nlm.nih.gov/nlmcatalog?term=Medicine%5Bst%5D) – includes General Medicine, Medical Research, Acta Medica and State Medical Journals |
| Allergy | [Allergy and Immunology](http://www.ncbi.nlm.nih.gov/nlmcatalog?term=Allergy%20and%20Immunology%5Bst%5D) – includes Hypersensitivity, Lymphology, Serology, Serotherapy, and Interferons |
| Cardiology | [Cardiology](http://www.ncbi.nlm.nih.gov/nlmcatalog?term=Cardiology%5Bst%5D) |
|  | [Vascular Diseases](http://www.ncbi.nlm.nih.gov/nlmcatalog?term=Vascular%20Diseases%5Bst%5D) – includes Blood Circulation, Hypertension, and Thrombosis |
| Critical Care | [Critical Care](http://www.ncbi.nlm.nih.gov/nlmcatalog?term=Critical%20Care%5Bst%5D) – includes Intensive Care |
| Endocrinology | [Endocrinology](http://www.ncbi.nlm.nih.gov/nlmcatalog?term=Endocrinology%5Bst%5D) – includes Hormones and Diabetes Mellitus |
|  | [Metabolism](http://www.ncbi.nlm.nih.gov/nlmcatalog?term=Metabolism%5Bst%5D) – includes Obesity |
| Gastroenterology | [Gastroenterology](http://www.ncbi.nlm.nih.gov/nlmcatalog?term=Gastroenterology%5Bst%5D) – includes Diseases of the Digestive System (Esophagus, Liver, Gallbladder, and Pancreas) |
| Geriatrics | [Geriatrics](http://www.ncbi.nlm.nih.gov/nlmcatalog?term=Geriatrics%5Bst%5D) – includes Aging and the Aged |
| Infectious Disease | [Acquired Immunodeficiency Syndrome](http://www.ncbi.nlm.nih.gov/nlmcatalog?term=Acquired%20Immunodeficiency%20Syndrome%5Bst%5D) |
|  | [Anti-Infective Agents](http://www.ncbi.nlm.nih.gov/nlmcatalog?term=Anti-Infective%20Agents%5Bst%5D) – includes Antibacterial, Antifungal, Antiviral, and Antiparasitic Agents |
|  | [Bacteriology](http://www.ncbi.nlm.nih.gov/nlmcatalog?term=Bacteriology%5Bst%5D) |
|  | [Parasitology](http://www.ncbi.nlm.nih.gov/nlmcatalog?term=Parasitology%5Bst%5D) – includes Protozoology |
|  | [Virology](http://www.ncbi.nlm.nih.gov/nlmcatalog?term=Virology%5Bst%5D) |
| Oncology | [Antineoplastic Agents](http://www.ncbi.nlm.nih.gov/nlmcatalog?term=Antineoplastic%20Agents%5Bst%5D) |
|  | [Neoplasms](http://www.ncbi.nlm.nih.gov/nlmcatalog?term=Neoplasms%5Bst%5D) – includes Medical and Experimental Oncology |
| Nephrology | [Nephrology](http://www.ncbi.nlm.nih.gov/nlmcatalog?term=Nephrology%5Bst%5D) – includes Dialysis and Hemodialysis |
| Primary Care | [Primary Health Care](http://www.ncbi.nlm.nih.gov/nlmcatalog?term=Primary%20Health%20Care%5Bst%5D) – includes Family Practice |
| Pulmonology | [Pulmonary Medicine](http://www.ncbi.nlm.nih.gov/nlmcatalog?term=Pulmonary%20Medicine%5Bst%5D) – includes Respiratory Tract Diseases, Thoracic Diseases, and Pulmonary Tuberculosis |
| Rheumatology | [Rheumatology](http://www.ncbi.nlm.nih.gov/nlmcatalog?term=Rheumatology%5Bst%5D) – includes Arthritis |

**Supplementary Table 2: National Library of Medicine (NLM) Broad Subject Terms included in each medical specialty.** In our work, we define a set of 13 medical specialties that include academic journals associated with specific Broad Subject Terms for Indexed Journals made available by the NLM. Broad Subject Terms “are assigned by NLM to MEDLINE journals to describe the journal's overall scope” according to the NLM.^9^

|  | **Reference Annotation Source** | | |
| --- | --- | --- | --- |
| **Gender Annotation Method** | **Human Annotator 1** | **Human Annotator 2** | **Human Annotators (Consensus)** |
| Gender Guesser^10^ | 0.337 | 0.438 | 0.339 |
| GenderizeR^11^ | 0.473 | 0.499 | 0.516 |
| Global Gender Predictor^12^ | 0.873 | 0.913 | 0.930 |
| Gender It^13^ | 0.842 | 0.870 | 0.884 |
| Gender Extractor^14^ | 0.803 | 0.855 | 0.884 |
| DameGender^15^ | 0.818 | 0.833 | 0.863 |
| GenderAPI^16^ | 0.870 | 0.923 | 0.945 |
| Meta Llama-3.1 8B^17^ (Single) | 0.878 ± 0.007 | 0.906 ± 0.005 | 0.957 ± 0.004 |
| Meta Llama-3.1 8B (Consensus) | 0.924 | 0.935 | 0.967 |
| OpenAI GPT-OSS 20B^18^ (Single) | 0.830 ± 0.008 | 0.903 ± 0.003 | 0.924 ± 0.008 |
| OpenAI GPT-OSS 20B (Consensus) | 0.884 | 0.947 | 0.966 |
| OpenAI GPT-OSS 120B^18^ (Single) | 0.669 ± 0.011 | 0.718 ± 0.011 | 0.724 ± 0.007 |
| OpenAI GPT-OSS 120B (Consensus) | 0.842 | 0.889 | 0.916 |
| Human Annotator 1 | 1.000 | 0.945 | 1.000 |
| Human Annotator 2 | 0.945 | 1.000 | 1.000 |
| Human Annotators (Consensus) | 1.000 | 1.000 | 1.000 |

**Supplementary Table 3: Evaluation of the agreement between automated gender annotation strategies and reference human annotations.** We show the Cohen’s kappa score that quantifies the inter-rater agreement between the reference human annotations and each of the automated gender annotation strategies. “Consensus” refers to the threshold-based algorithm of deriving a consensus gender label from multiple independent annotations, as described in the **Predicting the Gender of Manuscript Authors Using a Large Language Model** subsection in the **Materials and Methods** in the main text; we report results using τ = 2 (out of 3) for the database methods and τ = 4 (out of 5) for the LLM-based methods. “Single” refers to using only a single prediction for data labelling (i.e., ablating the consensus algorithm). Error values represent the standard error of the mean (SEM). The method used in the main text and in our subsequent supplementary experiments is underlined.

| **Failure Mode** | **Frequency (%)** | **Examples** | **Description** |
| --- | --- | --- | --- |
| Out-of-Distribution | 74.0 | Foo Chai | The first name is under-represented in the US, and so the Llama-3.1 8B LLM from Meta AI (a company in the US) is unlikely to correctly predict the gender. |
|  |  | 新利 |  |
|  |  | Nkoke |  |
| Unisex | 13.1 | Jamie | The first name is gender-neutral and can be traditionally associated with both females and males. |
|  |  | Andy |  |
|  |  | Taylor |  |
| Initials | 11.4 | H. A. W. | The first name consists of the author’s first initial and possible middle initial(s). |
|  |  | G R |  |
|  |  | M.A.S. |  |
| String Encoding Error | 1.2 | Ren√©e (Renée) | The encoding used by the NLM Entrez API results in garbled text and misrepresentations of the first name. |
|  |  | Christian¬†Z (Christian Z) |  |
|  |  | Aim√© (Aimé) |  |
| Organization | 0.3 | SIMBA and CoMICs | The first name is the name or abbreviation of an organization, and not a singular individual. |
|  |  | START2-POST-VTE |  |
|  |  | STRATOS |  |

**Supplementary Table 4: Failure modes of the LLM-based gender annotation pipeline.** We describe the observed failure modes of our pipeline (i.e., the “Meta Llama-3.1 8B (Consensus)” pipeline in **Supplementary Table 3** and in **Figure 2**) when it failed to assign a gender to a first name for 1000 randomly selected examples from our dataset, report their respective frequencies, and list 3 examples of each failure mode. In the “String Encoding Error” row, we also provide the predicted correct representation of each example name in parentheses; the predicted correct representation was generated by encoding the original name in Mac OS Roman and then decoding it back into UTF-8, recovering the original characters when possible.

|  | **F/F Count (%)** | **F/M Count (%)** | **M/F Count (%)** | **M/M Count (%)** | **Total (%)** |
| --- | --- | --- | --- | --- | --- |
| Medicine | 86,605 (19.1) | 102,078 (22.5) | 53,830 (11.9) | 130,964 (28.9) | 373,477 (82.5) |
| Allergy | 19,168 (19.7) | 23,563 (24.3) | 11,512 (11.9) | 24,506 (25.2) | 78,749 (81.1) |
| Cardiology | 7,873 (12.2) | 13,531 (21.0) | 6,835 (10.6) | 26,194 (40.6) | 54,433 (84.3) |
| Critical Care | 2,300 (12.8) | 3,656 (20.3) | 1,964 (10.9) | 7,680 (42.6) | 15,600 (86.5) |
| Endocrinology | 8,059 (20.1) | 9,533 (23.8) | 4,343 (10.9) | 10,326 (25.8) | 32,261 (80.6) |
| Gastroenterology | 5,992 (14.1) | 9,379 (22.1) | 4,378 (10.3) | 14,980 (35.2) | 34,729 (81.7) |
| Geriatrics | 10,425 (28.6) | 8,528 (23.4) | 4,595 (12.6) | 7,485 (20.5) | 31,033 (85.1) |
| Infectious  Disease | 6,416 (17.5) | 9,807 (26.7) | 4,288 (11.7) | 10,013 (27.3) | 30,524 (83.2) |
| Nephrology | 3,833 (16.7) | 5,467 (23.8) | 2,417 (10.5) | 7,879 (34.3) | 19,596 (85.4) |
| Oncology | 36,733 (18.4) | 43,893 (21.9) | 22,292 (11.1) | 55,597 (27.8) | 158,515 (79.2) |
| Primary Care | 6,147 (32.2) | 3,721 (19.5) | 2,268 (11.9) | 5,102 (26.7) | 17,238 (90.2) |
| Pulmonology | 6,578 (14.3) | 9,597 (20.8) | 4,911 (10.6) | 16,895 (36.6) | 37,981 (82.3) |
| Rheumatology | 3,818 (22.9) | 3,814 (22.8) | 2,108 (12.6) | 4,276 (25.6) | 14,016 (83.9) |
| **Total** | **203,947 (18.7)** | **246,567 (22.6)** | **125,741 (11.5)** | **321,897 (29.4)** | **898,152 (82.2)** |

**Supplementary Table 5: Distribution of manuscripts stratified by predicted genders of first and last authors.** We stratify the total number of manuscripts in our dataset based on the LLM-predicted genders of the first and last authors. Percentages are with respect to the total number of manuscripts—including those where either the gender of the first and/or last author could not be determined. The column “**[First]/[Last] Count**” refers to the number of manuscripts published in the journal with a predicted first author gender of [First] and last author gender of [Last], where **M** corresponds to male and **F** to female. Note that the percentages do not necessarily sum to 100% in the **Total Count** column, as some manuscripts exist where the gender of the first and/or last author could not be determined.

|  | **Journal** | **SJR (2024)** | **Manuscript Count (%)** | **Author Count (%)** | |
| --- | --- | --- | --- | --- | --- |
|  |  |  |  | **Female** | **Male** |
| **Medicine** | PloS One | 0.80 | 90,997 (20.1) | 279,132 (39.2) | 372,084 (52.3) |
|  | Medicine | 0.47 | 50,284 (11.1) | 247,037 (40.1) | 303,691 (49.3) |
|  | BMJ Open | 1.02 | 32,772 (7.24) | 131,269 (48.6) | 115,914 (42.9) |
| **Allergy** | Frontiers in Immunology | 1.94 | 39,868 (41.1) | 142,459 (44.5) | 138,474 (43.3) |
|  | Human Vaccines & Immunotherapeutics | 0.90 | 4,914 (5.06) | 14,833 (45.3) | 13,821 (42.2) |
|  | Infection and Immunity | 1.05 | 3,561 (3.67) | 11,350 (42.7) | 12,845 (48.4) |
| **Cardiology** | Journal of the American Heart Association | 2.19 | 9,409 (14.6) | 36,154 (32.2) | 63,399 (56.5) |
|  | BMC Cardiovascular Disorders | 0.71 | 4,859 (7.53) | 12,797 (35.4) | 17,364 (48.0) |
|  | Cardiovascular Diabetology | 3.35 | 2,610 (4.04) | 9,795 (36.9) | 13,270 (50.0) |
| **Critical Care** | Critical Care | 2.74 | 5,192 (28.8) | 26,725 (30.9) | 43,786 (50.6) |
|  | American Journal of Respiratory and Critical Care Medicine | 5.08 | 4,155 (23.0) | 10,965 (35.3) | 18,613 (60.0) |
|  | Critical Care Medicine | 2.12 | 1,379 (7.65) | 3,885 (34.8) | 6,747 (60.4) |
| **Endocrin-ology** | Oxidative Medicine and Cellular Longevity | 1.67 | 5,768 (14.4) | 18,443 (42.8) | 16,996 (39.4) |
|  | Redox Biology | 3.37 | 3,158 (7.89) | 12,183 (41.7) | 12,598 (43.2) |
|  | Journal of Clinical Endocrinology & Metabolism | 2.18 | 3,010 (7.52) | 14,666 (48.6) | 13,451 (44.6) |
| **Gastro-enterology** | World Journal of Gastroenterology | 1.42 | 7,553 (17.8) | 18,351 (36.2) | 25,188 (49.7) |
|  | BMC Gastroenterology | 0.84 | 2,337 (5.50) | 12,718 (34.3) | 18,480 (49.8) |
|  | Hepatology Communications | 2.11 | 1,813 (4.26) | 7,275 (39.3) | 9,752 (52.7) |
| **Geriatrics** | Geriatrics | 0.67 | 4,594 (12.6) | 28,952 (50.6) | 21,869 (38.3) |
|  | Journal of Alzheimer’s Disease | 1.17 | 2,563 (7.03) | 9,863 (45.6) | 10,523 (48.6) |
|  | Journal of the American Geriatrics Society | 1.89 | 2,515 (6.90) | 9,823 (54.8) | 7,308 (40.7) |
| **Infectious Disease** | Viruses | 1.15 | 14,659 (40.0) | 52,225 (45.5) | 53,943 (47.0) |
|  | Journal of Virology | 1.28 | 7,936 (21.6) | 26,794 (40.2) | 32,450 (48.7) |
|  | Virology Journal | 0.99 | 2,412 (6.58) | 8,292 (41.9) | 8,324 (42.1) |
| **Nephrology** | BMC Nephrology | 0.81 | 4,261 (18.6) | 13,965 (38.8) | 16,975 (47.2) |
|  | Clinical Journal of the American Society of Nephrology | 2.38 | 2,709 (11.8) | 6,889 (39.1) | 9,724 (55.2) |
|  | Journal of the American Society of Nephrology | 3.75 | 2,682 (11.7) | 9,217 (37.3) | 13,980 (56.6) |
| **Oncology** | Neuro-oncology | 6.97 | 17,943 (8.97) | 59,423 (35.1) | 83,887 (49.6) |
|  | Oncotarget | 0.79 | 16,427 (8.21) | 56,322 (39.0) | 66,949 (46.4) |
|  | Cancer | 2.95 | 7,830 (3.91) | 51,528 (39.1) | 60,319 (45.8) |
| **Primary Care** | British Journal of General Practice | 1.37 | 4,171 (21.8) | 6,371 (47.0) | 6,509 (48.0) |
|  | Annals of Family Medicine | 1.11 | 3,218 (16.8) | 9,448 (59.2) | 5,827 (36.5) |
|  | Atencion Primaria | 0.43 | 1,440 (7.54) | 3,220 (53.6) | 2,591 (43.1) |
| **Pulmon-ology** | BMC Pulmonary Medicine | 0.89 | 3,682 (7.98) | 11,289 (37.8) | 14,143 (47.3) |
|  | Journal of Cardiothoracic Surgery | 0.49 | 3,540 (7.67) | 5,625 (26.1) | 12,626 (58.5) |
|  | Thoracic Cancer | 0.80 | 3,059 (6.63) | 8,057 (31.2) | 13,253 (51.3) |
| **Rheumat-ology** | Rheumatology | 1.72 | 5,332 (31.9) | 39,733 (37.8) | 52,482 (49.9) |
|  | Arthritis Research & Therapy | 1.59 | 2,769 (16.6) | 11,368 (40.9) | 13,571 (48.9) |
|  | Pediatric Rheumatology Online Journal | 0.91 | 1,328 (7.95) | 9,530 (41.8) | 6,858 (30.1) |

**Supplementary Table 6: High-volume journals by medical subspecialty.** We report the 3 journals with the greatest number of unique manuscripts published between January 2015 and September 2025 within each specialty. The “Manuscript Count” percentage is the percentage of manuscripts published by the journal within the specialty. The “Author Count” percentages are the percentages of female and male authors publishing within each journal; note that they do not necessarily sum to 100% in each row, as the gender of some manuscript authors could not be determined.

|  | **Journal** | **SJR (2024)** | **Manuscript Count (%)** | **Author Count (%)** | |
| --- | --- | --- | --- | --- | --- |
|  |  |  |  | **Female** | **Male** |
| **Medicine** | New England Journal of Medicine | 19.1 | 1,618 (0.36) | 9,809 (35.2) | 12,362 (44.3) |
|  | Nature Medicine | 18.3 | 1,709 (0.38) | 20,296 (40.5) | 26,500 (52.9) |
|  | Nature Reviews Disease Primers | 18.0 | 97 (0.02) | 285 (38.1) | 440 (58.7) |
| **Allergy** | Nature Reviews Immunology | 17.5 | 463 (0.48) | 495 (37.2) | 771 (57.9) |
|  | Annual Review of Immunology | 16.4 | 75 (0.08) | 99 (41.3) | 137 (57.1) |
|  | Immunity | 12.2 | 1,161 (1.20) | 6,261 (38.5) | 8,715 (53.6) |
| **Cardiology** | Circulation | 8.67 | 2,290 (3.55) | 9,527 (32.4) | 18,156 (61.7) |
|  | JACC: Cardiovascular Imaging | 5.43 | 393 (0.61) | 1,642 (28.9) | 3,772 (66.5) |
|  | Circulation Research | 4.90 | 1,838 (2.85) | 5,517 (36.4) | 8,467 (55.9) |
| **Critical Care** | American Journal of Respiratory and Critical Care Medicine | 5.08 | 4,155 (23.0) | 10,965 (35.3) | 18,613 (60.0) |
|  | Intensive Care Medicine | 5.00 | 1,179 (6.54) | 12,770 (38.0) | 17,554 (52.2) |
|  | Critical Care | 2.74 | 5,192 (28.8) | 26,725 (30.9) | 43,786 (50.6) |
| **Endocrin-ology** | Cell Metabolism | 11.9 | 994 (2.48) | 4,857 (38.0) | 6,864 (53.8) |
|  | Nature Metabolism | 7.53 | 514 (1.28) | 3,389 (39.7) | 4,506 (52.8) |
|  | Bone Research | 4.05 | 452 (1.13) | 1,790 (38.4) | 2,003 (42.9) |
| **Gastro-enterology** | The Lancet Gastroenterology & Hepatology | 11.4 | 237 (0.56) | 2,359 (38.3) | 3,410 (55.4) |
|  | Journal of Hepatology | 10.6 | 687 (1.62) | 3,349 (36.7) | 5,135 (56.3) |
|  | Nature Reviews Gastroenterology and Hepatology | 9.91 | 214 (0.50) | 321 (33.1) | 594 (61.2) |
| **Geriatrics** | Nature Aging | 7.08 | 331 (0.91) | 2,149 (39.0) | 2,578 (46.7) |
|  | The Lancet Healthy Longevity | 5.17 | 173 (0.47) | 1,010 (42.2) | 1,137 (47.5) |
|  | Ageing Research Reviews | 4.22 | 212 (0.58) | 440 (37.4) | 622 (52.9) |
| **Infectious Disease** | Nature Microbiology | 6.89 | 903 (2.46) | 5,696 (39.9) | 7,600 (53.3) |
|  | The Lancet Microbe | 5.26 | 430 (1.17) | 2,968 (43.5) | 3,282 (48.1) |
|  | Annual Review of Virology | 4.03 | 74 (0.20) | 97 (39.9) | 119 (49.0) |
| **Nephrology** | Nature Reviews Nephrology | 7.31 | 251 (1.09) | 446 (36.1) | 745 (60.2) |
|  | Kidney International | 4.13 | 832 (3.63) | 3,865 (38.0) | 5,511 (54.2) |
|  | Journal of the American Society of Nephrology | 3.75 | 2,682 (11.7) | 9,217 (37.3) | 13,980 (56.6) |
| **Oncology** | CA: A Cancer Journal for Clinicians | 145 | 98 (0.05) | 423 (48.8) | 420 (48.5) |
|  | Nature Reviews Clinical Oncology | 28.7 | 180 (0.09) | 387 (35.9) | 651 (60.4) |
|  | Nature Reviews Cancer | 24.4 | 208 (0.10) | 300 (32.3) | 577 (62.2) |
| **Primary Care** | British Journal of General Practice | 1.37 | 4,171 (21.8) | 6,371 (47.0) | 6,509 (48.0) |
|  | Family Process | 1.33 | 248 (1.30) | 666 (61.2) | 353 (32.4) |
|  | Family Medicine and Community Health | 1.15 | 237 (1.24) | 719 (50.8) | 610 (43.1) |
| **Pulmon-ology** | Journal of Thoracic Oncology | 7.95 | 578 (1.25) | 2,460 (34.5) | 3,477 (48.7) |
|  | The Lancet Respiratory Medicine | 6.78 | 744 (1.61) | 5,608 (31.1) | 8,021 (44.6) |
|  | The European Respiratory Journal | 4.50 | 1,333 (2.89) | 6,316 (37.1) | 9,608 (56.5) |
| **Rheumat-ology** | Nature Reviews Rheumatology | 5.51 | 160 (0.96) | 304 (41.2) | 402 (54.5) |
|  | The Lancet Rheumatology | 3.84 | 340 (2.03) | 3,572 (45.8) | 3,781 (48.5) |
|  | Arthritis and Rheumatology | 3.78 | 1,277 (7.64) | 7,445 (42.4) | 8,235 (46.8) |

**Supplementary Table 7: High-impact journals by medical subspecialty.** We report the 3 journals with the highest 2024 SCImago Journal Rank (SJR, a measure of journal impact) score within each specialty. The “Manuscript Count” percentage is the percentage of manuscripts published by the journal within the specialty. The “Author Count” percentages are the percentages of female and male authors publishing within each journal; note that they do not necessarily sum to 100% in each row, as the gender of some manuscript authors could not be determined.

| **Medical Specialty** | **Single-Author Manuscript Count (%)** | **Single-Author Manuscript Counts by Gender (%)** | |
| --- | --- | --- | --- |
|  |  | **Female** | **Male** |
| Medicine | 18,479 (3.80) | 5,667 (30.7) | 10,350 (56.0) |
| Allergy | 2,741 (2.61) | 891 (32.5) | 1,686 (61.5) |
| Cardiology | 2,374 (3.52) | 475 (20.0) | 1,773 (74.7) |
| Critical Care | 1,088 (5.84) | 236 (21.7) | 776 (71.3) |
| Endocrinology | 1,193 (2.74) | 243 (20.4) | 652 (54.7) |
| Gastroenterology | 1,535 (3.43) | 421 (27.4) | 997 (65.0) |
| Geriatrics | 1,020 (2.64) | 376 (36.9) | 553 (54.2) |
| Infectious Disease | 884 (2.25) | 236 (26.7) | 562 (63.6) |
| Nephrology | 1,251 (5.26) | 407 (32.5) | 797 (63.7) |
| Oncology | 3,816 (1.75) | 1,145 (30.0) | 2,231 (58.5) |
| Primary Care | 3,724 (18.64) | 1,382 (37.1) | 2,162 (58.1) |
| Pulmonology | 1,917 (3.99) | 441 (23.0) | 1,361 (71.0) |
| Rheumatology | 409 (2.31) | 153 (37.4) | 202 (49.4) |
| **Total** | 40,431 (3.45) | 12,073 (29.9) | 24,102 (59.6) |

**Supplementary Table 8: Analysis of single-author manuscripts by medical specialty.** We report the total number of manuscripts authored by a single researcher in each medical field, and further stratify these single-author manuscripts in each row by the predicted gender of the author. Note that the sum of the female- and male- authored manuscripts in each row does not necessarily equal the total number of single-author manuscripts for that specialty, as the gender of some manuscript authors could not be determined using our LLM-based gender annotation pipeline. The percentage values in the second column correspond to the proportion of total manuscripts in the medical specialty that have a single author. The percentage values in the third and fourth columns correspond to the proportion of single-author manuscripts in the medical specialty authored by female and male authors, respectively.

# Supplementary Methods

**Large Language Model Prompt and Inference**

Our large language model (LLM) prompt used to predict the gender of an input name <|NAME|> is shown below. Newline characters are represented by the string \n. For phrase sets in blue text, exactly one phrase is sampled independently and randomly from the set with equal probability for each LLM query. In this way, there are 4 distinct LLM prompts that are used for the gender prediction task; however, note that these prompts are semantically invariant and only involved changing the order of the output options and the in-context examples.

Given a name, respond with whether the person is {male or female, female or male}.\n\n{Alice: female\n\nBob: male, Adam: male\n\nBella: female}\n\n<|NAME|>:

All experiments using LLMs were conducted using Hugging Face Transformers inference library (version 5.3.0) on a single NVIDIA RTX A6000 using 16-bit floating point quantization. For each query, we used a sampling temperature of 0.01, top-p value of 1.0, and a maximum number of new tokens to be generated of 1. All 5 independent predictions in the consensus pipeline used identical sampling parameters; stochasticity across runs arose solely from the randomized prompt phrasing described above using different random seeding values.

**Additional Evaluation of LLM Gender Annotation Pipeline**

In our main text, we describe how we benchmark our proposed LLM gender annotation method on publicly available datasets of name-gender pairs. To further characterize the performance of our approach specifically on the first names of manuscript authors, we constructed an additional “Human” benchmarking dataset where the ground-truth gender annotations were determined by human labelers. Firstly, 2 human annotators individually completed 2 hours of implicit bias training, and were then tasked with manually annotating the gender of a random subset of 1,000 unique author first names sampled across all 13 medical specialties. Each first name was annotated with exactly one of the following labels: “male,” “female,” or “unknown,” where “unknown” corresponds to cases of either unisex first names or when the annotator could not determine the gender. The annotators were only given access to author first names and could use any online tools to complete the task.

## Journal and Manuscript Metadata Curation

In our **Supplementary Results** and in **Supplementary Figures 6-7**, we sought to use our proposed LLM-based gender annotation approach to evaluate how author gender affects the scholarly impact of published manuscripts. To quantitatively measure a journal’s academic impact, we used the SCImago Journal Rank (SJR) published by the SCImago Research Group, which measures a journal’s citation impact over a 3-year period, where higher is better.^19^ For each manuscript published in a journal, we labelled a manuscript entry with the journal’s SJR from the year immediately prior to the publication year of the manuscript. The SJR metric can therefore be thought of as a proxy for the impact of an academic journal. We also recorded the Open Access (OA) status of each journal, which was also publicly provided by the SCImago Research Group; briefly, an OA journal generally publishes scholarly articles online for free, immediate access.

Finally, we wrote a separate custom Python script to query the OpenCitations API (v1.2.0) to obtain a manuscript’s total number of citations; number of “self-citations” (defined as a citation from a separate manuscript that shares at least one author, which is tracked in the OpenCitations database); and total number of references.^20^

## Cross-Cultural Performance of Gender Prediction Methods

We assessed the performance of eight gender prediction methods as a function of the geographic and cultural origin of names. Using the Global benchmarking dataset, we computed the per-country F1 score macro-averaged across male and female names, and then reported the average F1 score within each region weighted by the number of names in the benchmark from each country. Each country was assigned a corresponding region (one of Anglosphere, West/North Europe, South Europe, East Europe, Latin America, Caucasus/Central Asia, Middle East/North Africa, Sub-Saharan Africa, South Asia, Southeast Asia, East Asia, and Oceania); see **Supplementary Data 1** for the mapping between countries and regions. Of these regions, we grouped Anglosphere, West/North Europe, and South Europe as “Western” regions, and all other regions as non-Western regions.

# Supplementary Results

**Additional Evaluation of LLM Gender Annotation Pipeline**

In our **Supplementary Methods** section, we detail the construction of an additional “Human” benchmarking dataset consisting of a random sample of 1,000 unique first names of manuscript authors that were manually labelled by their corresponding gender according to two human annotators. The annotators achieved a Cohen’s kappa coefficient of κ = 0.945, indicating strong inter-rater reliability in manually labelling the gender of authors by first name. Restricting our analysis to the subset of first names where the two human annotators agreed on the final gender label, we found that our proposed LLM-based method achieved a Cohen’s kappa coefficient of κ = 0.967 with respect to the consensus labels between the human annotators (**Supplementary Table 3**). Furthermore, our approach also achieved an overall accuracy of 98.6%, and an accuracy of 98.5% (resp., 98.6%) on predicting the gender of female (resp., male) authors when compared to the reference human consensus labels.

Comparing our proposed approach to non-LLM-based annotation methods, we find that our method agrees more strongly with reference human annotations. Interestingly, we found that Meta Llama-3.1 8B subjectively exceeded the performance of the GPT-OSS models; this is likely because our input prompt described in the **Supplementary Methods** was optimized using the Meta Llama-3.1 8B model. Given that Meta Llama-3.1 8B already demonstrated impressive performance and can uniquely be run on existing consumer-grade hardware, we opted to use this model to power our experiments reported in the main text. Common failure modes of our final approach in failing to assign a gender to an author are reported in **Supplementary Table 4**. Future work might explore how to better adapt more powerful models and/or methods for bibliometric analyses.

Finally, we also examined the efficiency of our proposed method using Meta Llama-3.1 8B and our local hardware setup described in the **Supplementary Methods**; in our experiments, our LLM-based method was able to annotate author genders at a rate of 154.5 ± 7.1 msec per name (99.9% confidence interval), corresponding to approximately 4.3 hours to annotate 100,000 genders. We note that these results were obtained by running each of the $n=5$ LLM predictions sequentially; in theory, this rate could be substantially improved by parallelizing the independent LLM predictions across multiple GPUs or API calls, in addition to implementing more robust systems such as data caching and larger batch sizes.

## Within-Manuscript Gender Distribution of Author Lists

Understanding patterns of gender clustering within individual manuscript author lists may help elucidate whether researchers tend to publish and work with other researchers of the same (or different) genders. **Supplementary Figure 5** illustrates the distribution of proportions of female and male authors within author lists within a given manuscript. Restricting our attention to manuscripts with at least two authors, we found that research teams tended to fall into one of three configurations: primarily male, primarily female, or mixed gender with roughly equal representation of male and female researchers. Qualitatively, we observed that manuscripts were more likely to be primarily male than primarily female in Medicine, Cardiology, Critical Care, Gastroenterology, Infectious Disease, Nephrology, Oncology, and Pulmonology. In contrast, we observed the opposite trend in the fields of Geriatrics and Primary Care.

Within each medical specialty, we then ran a *z*-test for intra-group homophily under a random assignment model to quantitatively assess whether authors of the same gender tended to work together more frequently than what would be expected due to chance. The *p*-values across all specialties were each individually less than 10^-112^, suggesting a statistically significant trend consistent across all medical specialties that researchers of the same gender tend to work together as opposed to with researchers of the opposite gender. We found that the odds ratio (OR) of last authors working with a first author of the same gender as opposed to the opposite gender was 2.117 ± 0.031 (mean ± 99.9% CI) averaged across all specialties. Additional analysis included in **Supplementary Table 5**. Finally, we also analyze the gender-based distribution of manuscripts published in high-volume and high-impact journals in **Supplementary Tables 6** and **7**, respectively, and the distribution of single-author manuscripts in each medical discipline in **Supplementary Table 8**.

## Additional Analysis of Gender Impact on Author Lists

**Supplementary Table 5** stratifies the distribution of manuscripts based on the predicted genders of first and last authors. Cumulatively across all medical subspecialties, we found that the distribution of manuscripts was statistically unlikely to occur by chance, suggesting that first and last authors may preferentially choose to work with other authors based on gender (Pearson’s χ^2^ test: χ^2^ = 28524.851, df = 1, *p* = 0.0). However, Cramér’s V effect size is φ = 0.178 suggesting that the association is relatively small in magnitude. The observed odds ratio (OR) is 2.117 ± 0.031 (mean ± 99.9% CI), suggesting that last authors have about 2.117 times the odds of being paired with a first author of the same gender compared to with a first author of the opposite gender. Finally, we also computed the conditional probabilities P(First Author = male | Last Author = female) = 0.381, while P(First Author = female | Last Author = male) = 0.434, which suggests that both male and female last authors are more likely to have a first author of the same gender, with this tendency being more pronounced in male first- and last- author collaborations. We further stratify these results by medical specialty in the table below:

| **Specialty** | **χ^2^** | ***p*-value** | **φ** | **OR** | **P(M First \| F Last)** | **P(F First \| M Last)** |
| --- | --- | --- | --- | --- | --- | --- |
| Medicine | 11190.287 | 0.0 | 0.173 | 2.064 ± 0.047 | 0.383 | 0.438 |
| Allergy | 1366.684 | 3.65 x 10^-299^ | 0.132 | 1.732 ± 0.087 | 0.375 | 0.490 |
| Cardiology | 1704.858 | 0.0 | 0.177 | 2.230 ± 0.149 | 0.465 | 0.341 |
| Critical Care | 617.530 | 2.58 x 10^-136^ | 0.199 | 2.460 ± 0.316 | 0.461 | 0.323 |
| Endocrinology | 887.536 | 5.03 x 10^-195^ | 0.166 | 2.010 ± 0.162 | 0.350 | 0.480 |
| Gastroenterology | 1095.796 | 2.71 x 10^-240^ | 0.178 | 2.186 ± 0.178 | 0.422 | 0.385 |
| Geriatrics | 850.366 | 6.05 x 10^-187^ | 0.166 | 1.991 ± 0.162 | 0.306 | 0.533 |
| Infectious Disease | 305.385 | 2.21 x 10^-68^ | 0.100 | 1.528 ± 0.127 | 0.401 | 0.495 |
| Nephrology | 707.931 | 5.64 x 10^-156^ | 0.190 | 2.286 ± 0.249 | 0.387 | 0.410 |
| Oncology | 4864.171 | 0.0 | 0.175 | 2.087 ± 0.074 | 0.378 | 0.441 |
| Primary Care | 1677.467 | 0.0 | 0.312 | 3.716 ± 0.422 | 0.270 | 0.422 |
| Pulmonology | 1449.337 | 0.0 | 0.195 | 2.358 ± 0.184 | 0.427 | 0.362 |
| Rheumatology | 411.962 | 1.37 x 10^-91^ | 0.171 | 2.031 ± 0.249 | 0.356 | 0.471 |

## Manuscript Citation and Reference Counts

Citations and references constitute key indicators of scholarly impact.^21-25^ A citation refers to an instance where the manuscript is cited by a subsequent publication, whereas a reference is a previously published external work cited within the manuscript. We examined whether these metrics varied according to the gender of the first or last author. In **Supplementary Figure 6**, we found that manuscripts with male first or last authors generally demonstrated higher citation counts than those authored by females in corresponding positions, with statistically significant gender differences observed in 8 of the 13 evaluated medical subspecialties for first authors and 10 subspecialties for last authors. Male last authors were also associated with a greater number of “self-citations” per manuscript, which were defined as citations originating from publications sharing at least one author with the cited work. Although differences in reference counts between genders were occasionally statistically significant, we found that the absolute magnitude of these differences when averaged across all medical disciplines was relatively small at less than 1 reference for both the first and last author groups.

## Publication Journal Impact and Open Access Status

Finally, we examined the SCImago Journal Rank (SJR) of the journals that published articles written by first and last authors of different genders across different medical subspecialties. We observed a statistically significant difference between the mean SJR of journals publishing papers written by male and female first (resp., last) authors in 11 (resp., 10) out of 13 subspecialties (**Supplementary** **Fig. 7**). However, the absolute difference in mean SJR was relatively small in most cases; the average SJR associated with manuscripts published by male last authors was greater than that of female last authors by an absolute [relative] difference of 0.31 points [9.46%], 0.27 points [12.8%], 0.20 points [7.59%], and 0.20 points [10.6%] in the fields of Critical Care, Endocrinology, Allergy, and Nephrology, respectively. The observed mean gender-based difference in SJR was also greater as a function of the last author’s gender than as a function of the first author’s gender in all evaluated medical specialties except for Pulmonology. This is expected, as the last author (i.e., the Principal Investigator) is typically responsible for evaluating the suitability of a manuscript for a particular journal.

We also sought to evaluate the proportion of manuscripts published in Open Access (OA) journals as a function of publication year and gender. To this end, we estimated a generalized linear model with a binomial outcome using a logit link, with the proportion of OA manuscripts within each discipline as the dependent variable and gender, year, and their interaction as predictors. Our results suggest that manuscripts authored by female first or last authors were more likely to be published in OA journals when compared to their male counterparts in the fields of Gastroenterology, Infectious Disease, and Primary Care. In contrast, male first and last authors in Critical Care published more frequently in OA journals than female first and last authors, respectively. While there were some instances of subtle gender-based differences in the change in the proportion of OA manuscripts year over year, the differences by medical specialty were much more pronounced: the fields of Infectious Disease, Nephrology, and Primary Care were associated with the greatest increases in the proportion of OA manuscripts per year regardless of the genders of the first and last authors.

Of note, our citation and journal impact analyses were stratified by medical specialty and author position, and our Open Access analysis controls for publication year; however, additional covariates, such as including author career stage, institutional affiliation, country of origin, article type, and team size, were not modeled as potential confounders. Observed gender differences in citation counts and journal SJR should therefore be interpreted as descriptive associations rather than causal estimates, and future work incorporating author-level longitudinal metadata my help better isolate the independent contribution of gender from these other factors.

Ultimately, consistent with prior work, we found that clinical and medical research led by male senior PIs continue to receive more citations than those led by female senior PIs.^6,25,26^ Furthermore, the overall percentage of female first authors and senior authors has been only slowly increasing by an average of approximately 1% per year over the past decade (**Figure 3**). These findings collectively underscore ongoing disparities in authorship recognition and research practices in academic medicine.

## Distribution of Research Journals in Bibliometric Analysis

Traditional bibliometric analysis studies have limited the research scope to manuscripts published in a limited number of journals (**Supplementary Table 1**). In contrast, our work analyzes manuscripts from hundreds of research journals. To better assess the relative contributions of each journal to our overall analysis, we plot the number of manuscripts published per journal in **Supplementary Figure 1**; additional details are included in **Supplementary Data 2**. Within each medical subspecialty, we also list the 3 journals that published the greatest number of manuscripts between January 2015 and September 2025 in **Supplementary Table 6**, and the 3 journals with the highest impact (as estimated by the 2024 SCImago Journal Rank score) in **Supplementary Table 7**.

## Analysis of the Gender Distribution of Single-Author Manuscripts

Here, we analyze the gender-based distribution of manuscripts in each medical specialty that were authored by only a single individual. In **Supplementary Table 8**, we found that a total of approximately 3.45% of all manuscripts had a single author. The specialties with the greatest proportions of single-author manuscripts were Primary Care (18.64%), Critical Care (5.84%), and Nephrology (5.26%). In contrast, the fields of Oncology (1.75%), Infectious Disease (2.25%), and Rheumatology (2.31%) had lower proportions of single-author manuscripts.

There was also a statistically significant difference between the joint specialty-gender distribution of single-author manuscripts with that of all manuscript authors (Pearson’s χ^2^ test: χ^2^ = 36770.409, df = 25, *p* = 0.0). While the global Cramér’s V effect size was only φ = 0.062, we found there was a profound bias toward single authorship shared by both genders in Primary Care (Adjusted Standardized Residual (ASR) 81.020 for female authors and 156.963 for male authors), and there was a gender-independent bias against single authorship in Oncology (ASR -40.107 for female authors and 30.541 for male authors). We report all ASR values in the table below:

|  | **Adjusted Standardized Residuals (ASR) for Single Author Manuscripts** | |
| --- | --- | --- |
| **Specialty** | **Female** | **Male** |
| Medicine | -15.255 | 30.114 |
| Allergy | -15.445 | 3.233 |
| Cardiology | -10.552 | 12.525 |
| Critical Care | -2.981 | 16.587 |
| Endocrinology | -13.779 | 1.300 |
| Gastroenterology | -5.838 | 5.136 |
| Geriatrics | -12.156 | -3.519 |
| Infectious Disease | -12.746 | -0.666 |
| Nephrology | 7.794 | 21.991 |
| Oncology | -40.107 | -30.541 |
| Primary Care | 81.020 | 156.963 |
| Pulmonology | -3.756 | 21.255 |
| Rheumatology | -10.957 | -10.308 |

Of note, we observed that female authors were generally less likely to publish single-author manuscripts: 11 out of the 13 medical specialties were associated with a female ASR of less than -3.75. These results are consistent with prior work reporting under-representation of female researchers in single-author manuscripts across a variety of scientific disciplines.^27^

## Cross-Cultural Performance of Gender Prediction Methods

In general, we found that gender prediction methods generally struggled the most on East Asian and South Asian first names (**Supplementary Fig. 8a**). We found that the discrepancy in the performance between Western and non-Western names largely came down to these two regions. Even though database-based methods like Gender It and Global Gender Predictor demonstrated higher F1 scores than LLM-based methods (**Supplementary Fig. 8c**), our proposed method using both individual and consensus-based Llama-3.1-8B predictions was the only method to retain non-trivial performance across regions. However, our method still demonstrated male-favoring gender asymmetry in East Asia (**Supplementary Fig. 8b**).

# Supplementary References

1. Bruns N, Brensing P, Greve S, Horsch S, Felderhoff-Müser U, Dohna-Schwake C, Schwarz S. Female first and senior authorship in high-impact critical care journals 2005-2024. Crit Care 29(395): (2025). doi: 10.1186/s13054-025-05649-4
2. Brück O. A bibliometric analysis of the gender gap in the authorship of leading medical journals. Nat Commun Med 3(179): (2023). doi: 10.1038/s43856-023-00417-3
3. Böhme K, Klingelhöfer D, Groneberg DA, Bendels MHK. Gender disparities in pediatric research: A descriptive bibliometric study on scientific authorships. Ped Res 92: 80-90 (2022). doi: 10.1038/s41390-022-02010-1
4. Krstacic JE, Carr BM, Yaligar AR, Kuruvilla AS, Helali JS, Saragossi J, Zhu C, Hutnik R, Noubani M, Yang J, Tannous HJ, Shroyer ALW. Academic medicine’s glass ceiling: Author’s gender in top three medical research journals impacts probability of future publication success. PLoS One 17(4): e0261209 (2022). doi: 10.1371/journal.pone.0261209
5. Yao J, Nguyen AX, Xiang L, Li A, Wu AY. Geography, gender, and collaboration trends among global health authors. J Glob Health Rep 6: e2022014 (2022). doi: 10.29392/001c.33616
6. Chatterjee P, Werner RM. Gender disparity in citations in high-impact journal articles. JAMA Netw Open 4(7): e2114509 (2021). doi: 10.1001/jamanetworkopen.2021.14509
7. Campbell JC, Yoon SC, Grimm LJ. Authorship and impact of gender-specific research in major radiology journals. J Am Coll Radiol 16(2): 240-3 (2019). doi: 10.1016/j.jacr.2018.08.024
8. Jagsi R, Guancial EA, Worobey CC, Henault LE, Chang Y, Starr R, Tarbell NJ, Hylek EM. The “gender gap” in authorship of academic medical literature – A 35-year perspective. N Engl J Med 355(3): 281-7 (2006). doi: 10.1056/NEJMsa053910
9. U.S. National Library of Medicine. Broad subject terms for indexed journals. Accessed October 4, 2025. URL: https://journal-reports.nlm.nih.gov/broad-subjects
10. Elmas F. SexMachine (0.1.1) [Python Package]. Python Software Foundation (2013). Accessed September 6, 2025. URL: https://pypi.org/project/SexMachine
11. Wais, K. Gender prediction methods based on first names with genderizeR. The R Journal 8(1): 17-37 (2016). doi: 10.32614/RJ-2016-002
12. Klaver R. global-gender-predictor (0.0.5) [Python Package]. Python Software Foundation (2025). Accessed September 6, 2025. URL: https://pypi.org/project/global-gender-predictor
13. R4r_gender. Accessed March 4, 2026. URL: https://github.com/IES-platform/r4r_gender
14. Wydmański W. gender-extractor (0.1.3) [Python Package]. Python Software Foundation (2026). Accessed March 4, 2026. URL: https://pypi.org/project/gender-extractor
15. Menéndez DA. Un dataset internacional acerca de nombres, género y frecuencias en Damegender: Contando hombres y mujeres en Debian GNU/Linux. Revista Internacional de Aprendizaje 9(2): 75-95. (2023). doi: 10.18848/2575-5544/CGP/v09i02/75-95
16. GenderAPI. Accessed September 6, 2025. URL: https://www.genderapi.io
17. Grattafori A, Dubey A, Jauhri A, et al. The Llama 3 herd of models. arXiv Preprint (2024). doi: 10.48550/arXiv.2407.21783
18. OpenAI. gpt-oss-120b & gpt-oss-20b Model Card. arXiv Preprint (2025). doi: 10.48550/arXiv.2508.10925
19. Falagas ME, Kouranos VD, Arencibia-Jorge R, Karageorgopoulos DE. Comparison of SCImago journal rank indicator with journal impact factor. FASEB J 22(8): 2623-8. (2008). doi: 10.1096/fj.08-107938
20. Heibi I, Moretti A, Peroni S, Soricetti M. The OpenCitations Index: Description of a database providing open citation data. Scientometrics 129: 7923-42. (2024). doi: 10.1007/s11192-024-05160-7
21. Xiao N, Marquez-Karry R, de Oliveira DFM, Berggruen S, Horowitz JM. Gender disparities in academic radiology authorship: A 13-year review. Acad Radiol 30(8): 1714-20. (2023). doi: 10.1016/j.acra.2022.10.031
22. Piper CL, Scheel JR, Lee CI, Forman HP. Gender trends in radiology authorship: A 35-year analysis. Am J Roentgenol 206(1): 3-7. (2016). doi: 10.2214/AJR.15.15116
23. Hamidizadeh R, Jalal S, Pindiprolu B, et al. Influences for gender disparity in the radiology societies in North America. Am J Roentgenol 211(4): 831-8. (2018). doi: 10.2214/AJR.18.19741
24. Jutras M, Malekafzali L, Jung S, Das P, Qamar SR, Khosa F. National Institutes of Health: Gender differences in radiology funding. Acad Radiol 29(5): 748-54. (2022). doi: 10.1016/j.acra.2020.08.004
25. Goswami AK, Kokabi N, Khaja MS, et al. Academic radiology in the United States: Defining gender disparities in faculty leadership and academic rank. Acad Radiol 29(5): 714-25. (2022). doi: 10.1016/j.acra.2021.05.016
26. Xiao N, Mansukhani NA, de Oliveira DFM, Kibbe MR. Association of author gender with sex bias in surgical research. JAMA Surg 153(7): 663-70. (2018). doi: 10.1001/jamasurg.2018.0040
27. Holman L, Stuart-Fox D, Hauser CE. The gender gap in science: How long until women are equally represented? PLoS Biology 16(4): e2004956 (2018). doi: 10.1371/journal.pbio.2004956
